# Music Interventions for Older Adults in Clinical Trials and Research Studies Across Cultures and Settings

**DOI:** 10.1093/geroni/igaa057.3410

**Published:** 2020-12-16

**Authors:** Tara Rose, Elyse Manzo, Katherine Erickson, Joshua Valenzuela

**Affiliations:** University of Southern California, Los Angeles, California, United States

## Abstract

Music interventions and music therapy have become more common globally as nonpharmacological treatment options for memory loss, pain management, reduction of behavioral and psychological symptoms, and increased quality of life. Knowledge of multiethnic interventions is important when creating evidence-based programs within culturally diverse countries, such as the U.S. The purpose of this systematic review is to analyze music interventions for older adults across the globe to better understand emerging best practices. A review of all trials registered at clinicaltrials.gov and registries in the WHO Registry Network containing the key words “music therapy” were included, regardless of intervention type. Of the 627 studies generated, 449 met the eligibility criteria, with 11% enrolling only older adults and 89% enrolling older adults along with other age groups. Studies were conducted in 6 continents, 48 countries (23% in the U.S.), and in 23 languages. Music interventions for specific medical conditions (64%) or medical procedures (24%) were the primary foci in studies. While studies crossed multiple continents, less than 2% referenced ethnicity or culture in the study details. Detailed data on intervention types, demographics, measures, settings, and methodology will be presented. Results suggest that best practices in music therapy are being developed world-wide for the multitude of health challenges faced by older adults and demonstrate the diversity of music interventions in both medical and community settings. Information from this review can be used to improve the implementation of music intervention programs and may be particularly beneficial in countries with diverse multicultural populations.

